# Notes and News

**Published:** 1904-03-19

**Authors:** 


### Notes and News.

The Duchess of Albany will open the new Out-Patients' Department at the Richmond Hospital.

Mr. Burdett-Coutts, M.P., has resigned the office of Treasurer of the Great Northern Central Hospital.

Lady MacDonald has sent the Red Cross Hospital a donation of 1,740 yen, the proceeds of a concert held under her auspices, and Sir Claude MacDonald has also given 300 yen.

The National Health Society announces lectures on Infant Feeding at 4 p.m. on March 18 and 25, to be given by Dr. Robert Hutchison and Dr. Edmund Cautley, at the Cavendish Rooms, Mortimer Street. The first of the series of three lectures was delivered by Dr. Cautley on March 11.

The Board of Management of the Manchester Royal Infirmary have accepted a plan for the new infirmary designed by Mr. Edwin T. Hall and Mr. John Brooke, who will act as joint architects. This decision must be confirmed by the trustees. The infirmary, which is to contain about 600 beds, will be constructed on the pavilion principle. Thus it appears that the authorities will not adopt the retrograde scheme for a corridor hospital which at one time they contemplated.

The report issued by the National Association for the Prevention of Consumption shows that an increasing interest is being taken in all measures tending to the prevention of consumption throughout the country. It is not only in the erection of numerous sanatoria that this is evident, but by the adoption of sanitary measures and precautions apart from this. The effect of the numerous notices warning the public against the habit of spitting in public buildings and conveyances must tend to make an impression, and will probably serve equally well as a legal measure in educating the population.

One case of plague has occurred in Sydney.

It is proposed to hold a grand bazaar under Royal and distinguished patronage in aid of the funds of the Westminster Hospital in the summer of 1905.

The British Minister at Tokio having conveyed to the Japanese Minister of Marine Admiral Sir Cyprian Bridge's message offering the use of the British Naval Hospital at Yokohama for sick and wounded Japanese sailors and soldiers, the offer has been gratefully accepted.

The Edinburgh Local Government Board have made a recommendation to the governors of Poorhouses in Scotland to carefully examine the paupers applying for admission, with a view of securing vaccination or re-vaccination where advisable. The Board points out that the nomad population which frequents workhouses is one of the chief sources of infection. The wisdom of the recommendation is beyond dispute, but its compulsory adoption would be still more satisfactory.

In the report of the Liverpool School of Tropical Medicine, just issued, it is stated that the results of its teaching and operations are now beginning to appear, and that testimony is constantly arriving from West Africa and elsewhere in the tropics to the effect that the health conditions have been much improved where the recommendations of the school have been followed. During the year 31 students have attended the school, including officers of the Colonial Medical Service, the Indian Medical Service, the French Army Medical and the French Maritime Services, the Congo Free State Medical Service, and doctors and missionaries from countries as far apart as the Republic of Colombia and the Steppes of Tibet.

At a meeting to further the aims of the League of Mercy in Ealing, at which Mr. Leopold de Rothschild presided, he said, in asking the meeting to accord a hearty vote of thanks to the lecturer, he felt sure he was re-echoing their sentiments when he said that they had listened for an hour and a half with rapt attention to his marvellous description of the hospital work both in London and the provinces. He had told them, amongst other things, what was done in the hospitals, and he had asked them to support the hospitals, but he had not told them that he had given up his professional career, and the opportunity of making a great fortune, to speak and work on behalf of the funds of the League. Neither had he told them that the inception of the King Edward's Fund was his idea. Continuing, Mr. Rothschild said that every shilling collected by the League was given, without deduction, to the King's Fund. King Edward's Fund was administered with great care, and the money was not only given to the hospitals in accordance with their needs, but also in accordance with the way in which the hospital requiring assistance was administered. The shilling went, as it were, to ensure that the hospital was ably and carefully administered.

